# Supplementary material for: Genomic trends and emerging antimicrobial resistance in Neisseria gonorrhoeae over two decades in Kenya
Source: Microbiol Spectr. 2025 Oct 7;13(11):e01586-25. doi: 10.1128/spectrum.01586-25 (PMC12584697; doi:10.1128/spectrum.01586-25)
Supplement: Fig. S1 — Phylogenetic (maximum likelihood) tree based on the Ng cgMLST v2 scheme demonstrating alignment to the LIN code lineage and conventional typing schemes. [file spectrum.01586-25-s0001.docx]

**Supplemental Figure 1. Phylogenetic (Maximum Likelihood) Tree based on the Ng cgMLST v2 Scheme, Demonstrating Alignment to LIN code Lineage and Conventional Typing Schemes**

**
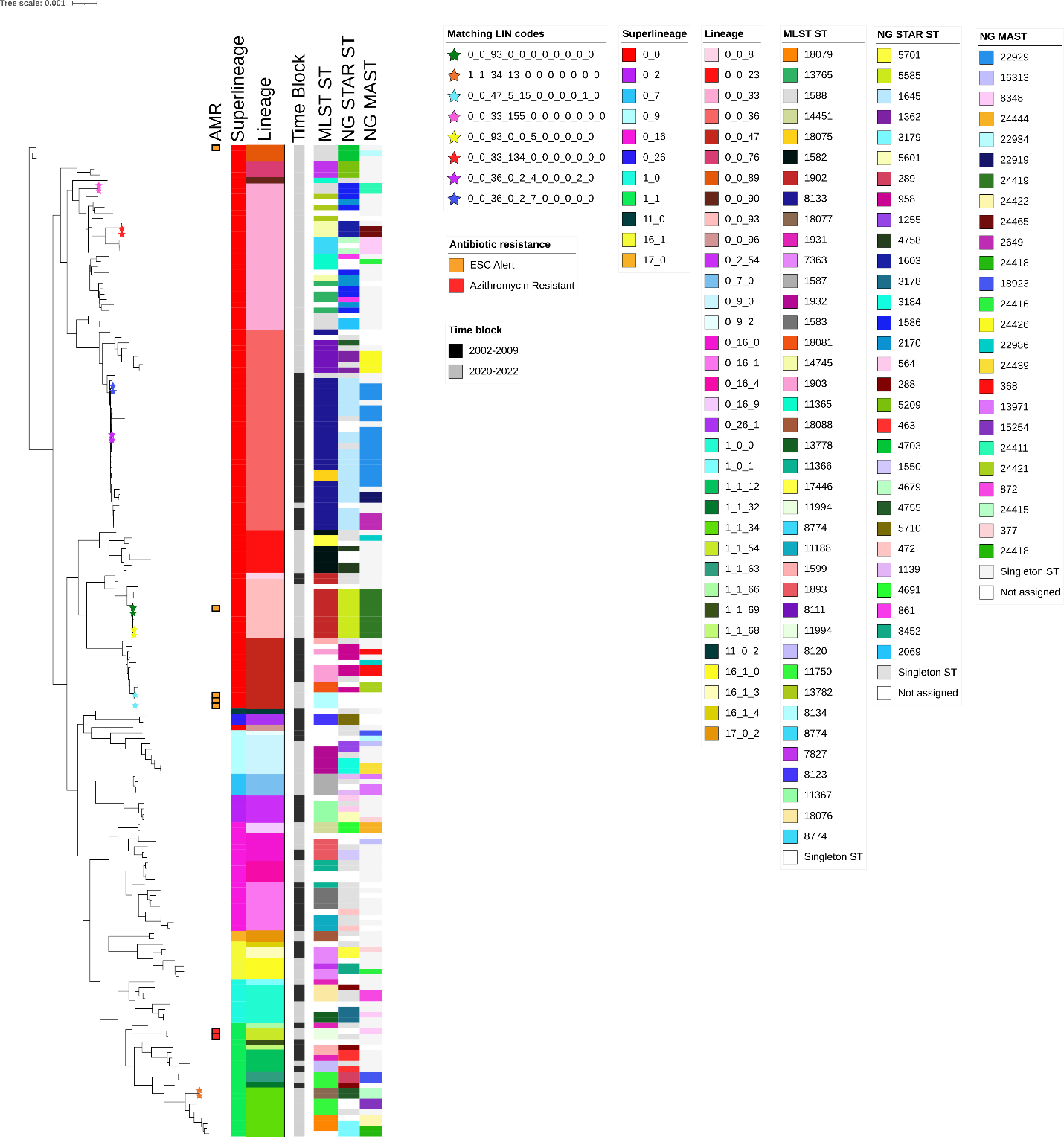
**

**Legend**: Supplemental Figure 1 depicts a maximum likelihood phylogenetic tree constructed based on 1430 core genes (Ng cgMLST v2), and shows the level of agreement between this phylogenetic clustering, LIN code lineage and conventional typing schemes such as 7-locus MLST. Each isolate is labelled with its year of isolation (Time Block), LIN code (superlineage and lineage), and its 7-locus MLST, NG STAR ST, and NG MAST ST. In the “AMR” column, the two isolates exhibiting azithromycin resistance are indicated with red blocks and the five isolates exhibiting cephalosporin alert values are indicated with orange blocks. Instances of matching full LIN codes, suggestive of highly related isolates, are indicated at the ends of branches with color coded stars. The tree was recombination corrected using ClonalframeML.
