# Supplementary material for: Genomic trends and emerging antimicrobial resistance in Neisseria gonorrhoeae over two decades in Kenya
Source: Microbiol Spectr. 2025 Oct 7;13(11):e01586-25. doi: 10.1128/spectrum.01586-25 (PMC12584697; doi:10.1128/spectrum.01586-25)
Supplement: Table S1 — List of genetic determinants and associated antimicrobial resistance or mechanism that was evaluated in the analysis. [file spectrum.01586-25-s0002.docx]

**Supplemental Table 1. List of genetic determinants and associated antimicrobial resistance or mechanism that was evaluated in the analysis.**

| Antimicrobial or  Mechanism | Determinant (if applicable: NEIS locus, NGO locus) |
| --- | --- |
| Penicillins | *bla*  *penA* D34  *penA* PBP2 (NEIS1753, NGO1542)  *mtrR* (NEIS1635, NGO1366) A39T, G45D  *porB1b* G120, A121  *porB/penB* (NEIS2020, NGO1812)  *ponA* (NEIS0414, NGO0099) L421P |
| Tetracyclines | Rpsj V57M  *mtrR* A39, G45  *porB* G120, A121  *ponA* L421P  *mtrR* promoter (*^pro^*NEIS1635)  *tetm* (NEIS2210) |
| Spectinomycin | 16S rRNA C1192U |
| Fluoroquinolones | *gyrA* (NEIS1320, NGO0629) S91F, D95N, D95G  *parC* (NEIS1525, NGO1259) D86N, S88P, E91K |
| Macrolides | 23S rRNA A2059G, C2611T  *mtrR* (NEIS1635, NGO1366) A39T, G45D  *mtrR* promoter (*^pro^*NEIS1635) -35Adel  *ermB*, *ermC* |
| Cephalosporins | *PenA a*lterations in: A311V, I312M, V316T, V316P, T483S, A501P, A501V, N512Y, G545S  *PenA* A501V, A501T  *Mtrr* A39, G45  *porB/penB* (NEIS2020, NGO1812) |
| Iron acquisition | *tbpB* (NEIS1690/1691) |
| Iron acquisition | *lbpB* (NEIS1468/1469) |
| Iron acquisition | *hpuA/hpuB* (NEIS1946/1947) |
| Iron acquisition | *fetA* (NEIS1963) |
| Iron acquisition | *fbpC* (NEIS004/1022) |
| Metabolism | *pykA* (NEIS0074) |
| Metabolism | *Ppk* (NEIS0323) |
| Metabolism | *sucD* (NEIS0925) |
| Metabolism | *sucD* (NEIS0936) |
| Housekeeping/  MLST | *abcZ*  *adk*  *aroE*  *fumC*  *gdh*  *pdhC*  *pgm* |
| Housekeeping | 16s rRNA T1458C |

References used for classifications in table

1. Unemo M, Shafer WM. Antimicrobial resistance in Neisseria gonorrhoeae in the 21st century: past, evolution, and future. Clinical microbiology reviews. 2014;27(3):587-613.

2. Harrison OB, Clemence M, Dillard JP, Tang CM, Trees D, Grad YH, Maiden MC. Genomic analyses of Neisseria gonorrhoeae reveal an association of the gonococcal genetic island with antimicrobial resistance. Journal of Infection. 2016;73(6):578-87.

3. Demczuk W, Martin I, Sawatzky P, Allen V, Lefebvre B, Hoang L, Naidu P, Minion J, VanCaeseele P, Haldane D. Equations to predict antimicrobial MICs in Neisseria gonorrhoeae using molecular antimicrobial resistance determinants. Antimicrobial Agents and Chemotherapy. 2020;64(3):10.1128/aac. 02005-19.

4. Cornelissen CN. Subversion of nutritional immunity by the pathogenic Neisseriae. Pathogens and disease. 2018;76(1):ftx112.

5. Diallo K, MacLennan J, Harrison OB, Msefula C, Sow SO, Daugla DM, Johnson E, Trotter C, MacLennan CA, Parkhill J. Genomic characterization of novel Neisseria species. Scientific reports. 2019;9(1):13742.
